# Molecular mechanisms driving homeostatic plasticity of neurotransmitter release

**DOI:** 10.3389/fncel.2013.00244

**Published:** 2013-12-03

**Authors:** Vesna Lazarevic, Santosh Pothula, Maria Andres-Alonso, Anna Fejtova

**Affiliations:** ^1^Department of Neurochemistry and Molecular Biology, Leibniz Institute for NeurobiologyMagdeburg, Germany; ^2^Research Group Presynaptic Plasticity, Leibniz Institute for NeurobiologyMagdeburg, Germany; ^3^Center for Behavioral Brain SciencesMagdeburg, Germany

**Keywords:** presynaptic homeostatic plasticity, probability of neurotransmitter release, presynaptic muting, cytomatrix at the active zone, ubiquitin proteasome system

## Abstract

Homeostatic plasticity is a process by which neurons adapt to the overall network activity to keep their firing rates in a reasonable range. At the cellular level this kind of plasticity comprises modulation of cellular excitability and tuning of synaptic strength. In this review we concentrate on presynaptic homeostatic plasticity controlling the efficacy of neurotransmitter release from presynaptic boutons. While morphological and electrophysiological approaches were successful to describe homeostatic plasticity-induced changes in the presynaptic architecture and function, cellular and molecular mechanisms underlying those modifications remained largely unknown for a long time. We summarize the latest progress made in the understanding of homeostasis-induced regulation of different steps of the synaptic vesicle cycle and the molecular machineries involved in this process. We particularly focus on the role of presynaptic scaffolding proteins, which functionally and spatially organize synaptic vesicle clusters, neurotransmitter release sites and the associated endocytic machinery. These proteins turned out to be major presynaptic substrates for remodeling during homeostatic plasticity. Finally, we discuss cellular processes and signaling pathways acting during homeostatic molecular remodeling and their potential involvement in the maladaptive plasticity occurring in multiple neuropathologic conditions such as neurodegeneration, epilepsy and neuropsychiatric disorders.

Homeostatic plasticity is a process by which neurons adapt to the overall network activity to keep their firing rates in a reasonable range. At the cellular level this kind of plasticity comprises modulation of cellular excitability and tuning of synaptic strength. In this review we concentrate on presynaptic homeostatic plasticity controlling the efficacy of neurotransmitter release from presynaptic boutons. While morphological and electrophysiological approaches were successful to describe homeostatic plasticity-induced changes in the presynaptic architecture and function, cellular and molecular mechanisms underlying those modifications remained largely unknown for a long time. We summarize the latest progress made in the understanding of homeostasis-induced regulation of different steps of the synaptic vesicle cycle and the molecular machineries involved in this process. We particularly focus on the role of presynaptic scaffolding proteins, which functionally and spatially organize synaptic vesicle clusters, neurotransmitter release sites and the associated endocytic machinery. These proteins turned out to be major presynaptic substrates for remodeling during homeostatic plasticity. Finally, we discuss cellular processes and signaling pathways acting during homeostatic molecular remodeling and their potential involvement in the maladaptive plasticity occurring in multiple neuropathologic conditions such as neurodegeneration, epilepsy and neuropsychiatric disorders.

Brain function is based on signal transmission between neurons assembled in complex networks. Structural and functional reorganization of these neuronal networks in processes generally termed neuronal plasticity underlie the cognitive performance of the brain including learning and memory. This plasticity is mediated by the modification of the signal processing within and between neurons of these networks. Associative or Hebbian plasticity induces changes in synaptic transmission, which are use-dependent and lead to reinforcement of active and weakening of inactive circuits. However, if acting repetitively Hebbian plasticity processes would lead to saturation or complete inactivation of synaptic function and in turn to functional destabilization of neuronal networks. Homeostatic plasticity acts to balance changes induced by Hebbian plasticity and ensures the maintenance of physiological network activity levels.

At the cellular level homeostatic plasticity comprises modulation of cellular excitability and tuning of synaptic strength by both pre- and postsynaptic mechanisms. Here, we focus on the discussion of homeostatic plasticity processes affecting neurotransmitter release from presynaptic boutons. The phenomenon of homeostatic adaptation of the presynaptic release machinery to the levels of ongoing activity was first described for the *Drosophila* neuromuscular junction (NMJ; Petersen et al., 1997; Davis et al., 1998; Davis and Goodman, 1998). Morphological and functional alterations of presynapses induced by global changes of network activity have also been reported in mammalian neurons more than a decade ago (Murthy et al., 2001). In the following years, a number of studies reported presynaptic homeostatic plasticity induced by various stimuli and using different experimental models, ranging from cultured dissociated neurons and cultured brain slices to intact animals (Bacci et al., 2001; Burrone et al., 2002; Desai et al., 2002; Moulder et al., 2004; Thiagarajan et al., 2005; Wierenga et al., 2006). Taken together, these studies revealed that presynaptic efficacy is elevated when levels of activity decrease, while neurotransmitter release at synapses is less efficient after overall increase of network activity.

Before describing how modulation of presynaptic efficacy occurs we briefly summarize the process of presynaptic neurotransmitter release. Neurotransmitter is stored in synaptic vesicles (SVs), which can release their content by controlled fusion with a specialized region of the presynaptic membrane named active zone (AZ). Central synapses contain around 200 SVs, which are not uniform regarding their functionality and localization. Different pools of vesicles have been described: the readily releasable pool (RRP, 5–9 vesicles), morphologically characterized by their physical contact with the AZ membrane, the recycling pool (RP) varies between 30 and 70% of all vesicles and contains SVs that can undergo exocytosis upon stimulation and resting pool (RtP) comprising vesicles that are incapable of exocytosis under physiological conditions (**Figure 1A**). Vesicles of the RRP are released within a few milliseconds to seconds during stimulation at 10–40 Hz (Stevens and Williams, 2007) or by the application of a hypertonic pulse of sucrose (Rosenmund and Stevens, 1996). Vesicles of RP are released upon prolonged stimulations, when RRP has been depleted. Together, RRP and RP form the total recycling pool (TRP). Vesicles of the RRP have the highest fusion probability of all vesicles. Therefore, the size of RRP is decisive for the synaptic release probability (Pr) and often assessed as a parameter of presynaptic strength (for comprehensive review on synaptic vesicle pools, SVP see Alabi and Tsien, 2012). Voltage-dependent calcium (Ca^2^^+^) channels (Cavs), which open in response to action potential-driven depolarization of the presynaptic membrane and mediate the Ca^2^^+^ influx into boutons, are crucial for evoked release. On the one hand they are regulated with respect to their localization within the AZ (Gundelfinger and Fejtova, 2012; Sudhof, 2012) and on the other hand through modification of their properties by multiple signaling pathways (Catterall and Few, 2008). Mechanistically, homeostatic changes in presynaptic Pr were mostly attributed to the modulation of the SVP (especially of the RRP; Murthy et al., 2001; Moulder et al., 2006), and to changes in action potential-induced Ca^2^^+^ influx (Moulder et al., 2003; Frank et al., 2006; Zhao et al., 2011; Muller and Davis, 2012).

**FIGURE 1:**
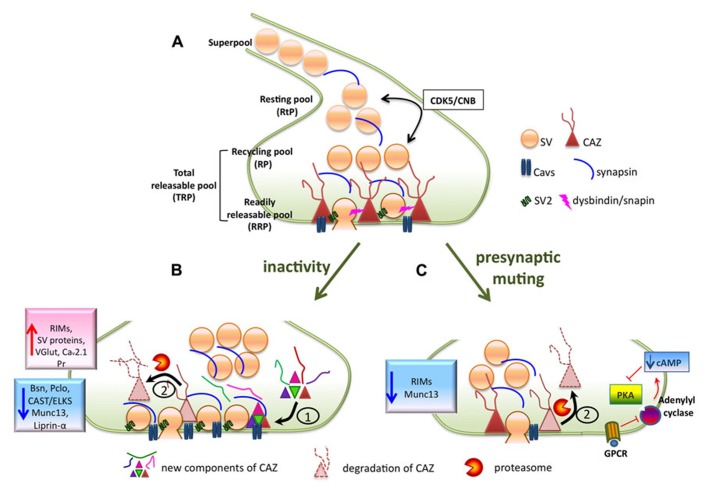
**(A)**Scheme of a presynaptic bouton containing synaptic vesicles that can be assigned to functionally different pools (RRP, RP, RtP), the release machinery (depicted as cytomatrix at the active zone, CAZ) and the presynaptic Ca^2^^+^ channels Cavs. **(B)** Prolonged activity deprivation induces extensive structural (enlargement of active zones) and functional (increased RRP and Pr) remodeling of presynaptic active zones, which is associated with a molecular reorganization of the release machinery. Protein synthesis **(1)** as well as protein degradation via the ubiquitin-proteasome system (UPS), **(2)** play a role in this process. **(C)** Depolarization-induced presynaptic muting is characterized by decreased size of RRP, depressed Pr and the reduction of CAZ proteins RIM and Munc13, probably due to their degradation by UPS. Induction/recovery from presynaptic muting also involves regulation of adenylate cyclase activity, which is an upstream regulator of PKA.

This review highlights the recent advances in our understanding of cellular mechanisms and identification of molecular players contributing to homeostatic modulation of the presynaptic Pr.

## PRESYNAPTIC HOMEOSTATIC PLASTICITY IN *Drosophila*

A comprehensive review of homeostatic plasticity on *Drosophila* NMJ was published recently (Frank, 2013). In this section, we will summarize the homeostatic modulation of presynaptic function with emphasis on identified molecular mechanisms acting during the adaptation of neurotransmitter release in this model system.

Early observations on homeostasis-induced modifications of synaptic transmission at the NMJ arose from developmental studies. During *Drosophila* larval development the surface area of muscles increase dramatically in a short period of time, decreasing the input resistance and evoking a growth of presynaptic nerve terminals and an increment in the number of boutons and AZs, resembling homeostatic adaptations. Genetic manipulations leading to an increased muscle innervation results in a compensatory, target-specific decrease in presynaptic transmitter release, whereas decreased muscle innervation results in a compensatory increase in quantal size (Davis and Goodman, 1998). Similarly, when the functionality of glutamate receptors was genetically ablated or the postsynaptic membrane was hyperpolarized by expression of Kir2.1 potassium channel, the presynaptic terminal responded by increasing the quantal content (i.e., increasing the number of presynaptic vesicles released per stimulus; Petersen et al., 1997; Davis et al., 1998; Paradis et al., 2001). This is well comparable with the later described increase of Pr at synapses of the vertebrate central nervous system (Murthy et al., 2001), implying evolutionary conservation of homeostatic plasticity mechanisms.

In contrast to vertebrate synapses (Plomp et al., 1992; Han and Stevens, 2009), the manifestation of presynaptic homeostatic plasticity is remarkably rapid in *Drosophila* larvae. Potentiation of presynaptic release appeared within 5–10 min upon application of philantotoxin-433 (PhTx), a persistent use-dependent antagonist of glutamate receptors (Frank et al., 2006). Both rapid induction and persistent expression of presynaptic homeostatic plasticity were blocked by mutations of Cav2.1, the pore-forming subunit of *Drosophila* calcium channel encoded by the *cacophony (cac)* gene. A recent publication by the same group, revealed ephexin, a Rho-type guanine nucleotide exchange factor, as indispensable for homeostasis-evoked increase of neurotransmitter release (Frank et al., 2009). Ephexin acts primarily with Cdc42 in a signaling system that converges on Cav2.1, further supporting its key importance during presynaptic homeostatic plasticity. Of note, PhTx-induced presynaptic homeostatic plasticity does not require new protein synthesis as it was shown for some forms of presynaptic homeostatic plasticity in vertebrates (Sutton et al., 2006; Han and Stevens, 2009). Nevertheless, fast molecular remodeling of presynaptic release apparatus occurs during this rapid functional plasticity as demonstrated, e.g., for the protein bruchpilot (Weyhersmuller et al., 2011), which is enriched at presynaptic release sites upon pharmacological blockage of glutamate receptors. Bruchpilot is a core component of AZ specializations at the NMJ, named T-bars, implied in the organization of Cav2.1 at release sites (Kittel et al., 2006); it might therefore contribute to the additional recruitment of functional Cav2.1 during homeostatic presynaptic strengthening.

Using calcium imaging, a recent study by Muller and Davis (2012) revealed alteration of presynaptic Ca^2^^+^ influx upon induction of presynaptic homeostatic plasticity. They suggested that modulation of Ca^2^^+^ influx is sufficient to account for the rapid induction and maintenance of a homeostatic change in vesicle release. In agreement with this claim their analyses did not reveal variations in the number of docked vesicles released by hypertonic sucrose solution. Important to note, assaying the RRP by an alternative method (i.e., measurement of amplitudes of cumulative excitatory postsynaptic currents and fluctuation analysis) revealed changes in a comparable experimental design (Weyhersmuller et al., 2011; Muller et al., 2012). An imaging study employing a genetically coded Ca^2^^+^ sensor revealed that increased Ca^2^^+^ influx determines the induction of homeostatic changes of neurotransmitter release also in murine cultured hippocampal neurons (Zhao et al., 2011). However, whether the modulation of Ca^2^^+^ influx is achieved by increasing the number of Cavs or by modifying their gating properties requires further studies.

Other molecules required for homeostatic increase of presynaptic strength at NMJs are dysbindin and snapin, both involved in calcium-dependent release of SVs (Dickman and Davis, 2009; Dickman et al., 2012). These proteins seem to act downstream or independently of Cav2.1. *Dysbindin*, a gene linked to schizophrenia in humans, was identified in a screen for genes involved in homeostatic modulation of presynaptic release. It is localized to the SV cluster and might play a role in regulation of SVP (Dickman and Davis, 2009). Snapin interacts and acts together with dysbindin in synchronizing calcium-dependent SV exocytosis by interacting with the t-SNARE protein SNAP25 during membrane trafficking events (**Figure 1**; Dickman et al., 2012). Importantly to state, the studies on dysbindin/snapin also suggested a potential link between homeostatic signaling and neurological disease.

From the same screen for proteins playing a role in presynaptic homeostatic plasticity *gooseberry,* a pair-rule transcription factor that antagonizes Wingless (*Drosophila* homologue of Wnt) signaling during development, was isolated (Marie et al., 2010). Gooseberry is required for sustained expression of synaptic homeostasis and it possibly links Wingless signaling to this process. Recently, it was also shown that the expression of miR-310-313 microRNA cluster is required for normal synaptic homeostasis. miR-310-313 binds to the 3’UTR of the kinesin motor family member Khc-73 and attenuates its expression. When mutated, Khc-73 expression rises, which is accompanied by higher expression of bruchpilot and increased numbers of T-bars in presynapses (Tsurudome et al., 2010) These studies indicate that regulation of gene expression and new protein synthesis might contribute to homeostatic processes at presynapse.

Rab3 and Rab3-interacting molecule (RIM), both well-described regulators of synaptic vesicle release, were recently also shown to be necessary for presynaptic homeostasis in *Drosophila*. Rab3, a small GTPase present on SVs, when bound to GTP seems to suppress synaptic homeostastic adapation at a very late stage of synaptic vesicle exocytosis. At the same time, the Rab3 GTPase activating protein (Rab3-GAP), which regulates this inhibitory effect by accelerating GTP hydrolysis to convert Rab3-GTP into Rab3-GDP, turns out to be essential for expression of homeostatic plasticity (Muller et al., 2011). RIM is an evolutionary conserved scaffolding protein of the presynaptic AZ. Apart from being important in the maintenance of basal synaptic transmission, RIM is required for the homeostatic plasticity-evoked increase in the RRP of SVs (Muller et al., 2012). The modulation of the RRP by RIM during homeostatic adaptation in *Drosophila* NMJ is a Ca^2^^+^-independent process what implies that RIM is not involved in the inactivity-induced modulation of Ca^2^^+^ influx through Cav2.1 in this model system (Muller et al., 2012).

## REGULATION OF SYNAPTIC VESICLE POOLS DURING HOMEOSTATIC PLASTICITY

In the pioneering work of Murthy et al. (2001) dealing with homeostatic plasticity in cultured hippocampal neurons it was demonstrated that changes supporting homeostatic scaling (inactivity-induced compensatory increase of quantal amplitude) were not only confined to postsynaptic terminals, as it was reported previously for the mammalian system (Turrigiano et al., 1998), but also affected the presynaptic machinery in a similar way as it was reported for the *Drosophila* NMJ. Imaging SV cycling via FM-dye loading showed that two days of activity blockade by interference either with action potential propagation using TTX or with AMPA receptor function by application of their antagonist NBQX led to an increase of the Pr and RRP size by about 50%. An electron microscopic analysis revealed an increase in the number of docked vesicles, the total number of vesicles in boutons and the area of AZs (**Figure 1**; Murthy et al., 2001). This study not only provided the first description of disuse-induced regulation of release in vertebrate neurons, but also supported the view that the RRP is formed by docked vesicles. A contrary approach, i.e., elevation of synaptic activity by depolarization, revealed a decrease in RRP (Moulder et al., 2006), and induced so-called synaptic muting, a phenomenon that will be discussed in detail below. Thus the RRP represents an important substrate for homeostatic plasticity and is bidirectionally regulated by overall activity levels to preserve stable firing rates. Multiple studies demonstrated that the total SVP and the TRP were also regulated during homeostatic adaptation to overall activity levels (Murthy et al., 2001; Thiagarajan et al., 2005; Branco et al., 2008; Lazarevic et al., 2011), nevertheless, the molecular players involved in this regulation remain poorly understood.

A key role in the regulation of the RtP during homeostatic plasticity was suggested recently for cyclin-dependent protein kinase 5 (CDK5; Kim and Ryan, 2010). The acute pharmacological inhibition of CDK5 activity resulted in an unmasking of previously silent synapses and in a mobilization of SVs of RtP resulting in the relative increase of the TRP. Neurons from CDK5 knock-out mice showed larger TRPs and in contrast to wild-type neurons, they did not further increase their TRP in response to inactivity. The regulation of CDK5 activity during inactivity occurs via regulation of protein turnover as inactivity led to a significant decrease in CDK5 expression levels. The protein phosphatase calcineurin B (CNB) seems to antagonize CDK5 in this regulation as neurons from CNB knock-out mice had a strongly reduced TRP. Thus the balance between CDK5 and CNB activity seems to determine the size of the RtP and the amount of vesicles available for action potential-driven release (**Figure 1A**). Nevertheless, the mechanism of CDK5 regulation during homeostatic plasticity remains to be addressed in future studies. Alterations of synaptic CDK5 amounts might be achieved by regulation at the level of gene expression, protein synthesis or degradation, which all reported to be involved in homeostatic plasticity-induced regulation of other protein targets. Regulation of CDK5 activity by dynamic association with its activator p25/p35 was shown in a recent study investigating the effects of activity withdrawal in hippocampal organotypic slices (Mitra et al., 2012) further confirming a key role of this kinase during homeostatic synaptic plasticity. However, the possible targets of CDK5 in this process are not known. Synapsin, a SV-associated protein, is phosphorylated by CDK5, which controls its recruitment to synapses (Easley-Neal et al., 2013) and the mobility of vesicles belonging to the so called “superpool” (Orenbuch et al., 2012). This is a population of SVs capable of traveling along axons and taking part in transmission at different presynaptic terminals (Krueger et al., 2003; Darcy et al., 2006; Westphal et al., 2008; Staras et al., 2010). Although more studies need to be performed to understand the function of the “superpool,” it seems likely that this mobile pool of vesicles can contribute to homeostatic adaptations (**Figure 1**), either by modulating SV pool sizes, mainly the RtP, or by acting as a readily accessible extrasynaptic pool (Staras and Branco, 2010).

Many further CDK5 substrates have been identified, which might potentially contribute to the modulation of presynaptic function during homeostatic adaptation; e.g., Munc18 (Fletcher et al., 1999), CASK (Samuels et al., 2007), or N-type Ca^2^^+^ channels (Su et al., 2012). However, the exact contribution of their modulation by CDK5 during homeostatic adaptations still needs to be elucidated.

The existence of the RtP and its contribution to the presynaptic plasticity was questioned in a recent study focusing on the CA3 to CA1 synapses in hippocampal slice cultures, where RtP could only be detected in immature (day *in vitro* 4) but not in mature (day *in vitro* 14–20) preparations (Rose et al., 2013). Nevertheless, strong depolarization in this system also led to the emergence of a release-incapable RtP. Similarly, pharmacological inhibition of CNB resulted in an increased RtP; this was, however, not statistically significant. Thus, it is possible that at mature CA1 to CA3 synapses the RtP emerges merely in reaction to pathophysiological situations such as stroke or seizures to reduce synaptic output and to prevent excitotoxicity.

## HOMEOSTATIC ADAPTATION OF VESICULAR FILLING

Besides altering the mode of SV release, homeostatic synaptic plasticity also comprises changes in the vesicular filling with neurotransmitters. At hippocampal synapses, glutamate receptors are not saturated by quantal release (McAllister and Stevens, 2000). Thus, changes in the amount of glutamate released from a single vesicle modulate the strength of glutamatergic neurotransmission. Studies in *Drosophila* and mammals revealed that the number and type of the vesicular transporter proteins present in the vesic membrane determine the amount and type of neurotransmitter loaded into SVs (Wojcik et al., 2004, 2006; Wilson et al., 2005; Daniels et al., 2006).

De Gois et al. (2005) demonstrated that mRNA and protein expression of glutamate transporters VGLUT1 and VGLUT2 as well as of the GABA transporter VIAAT/VGAT are regulated by activity levels in cultured neocortical cells. Withdrawal of network activity by application of TTX for 2days leads to an up-regulation of VGLUT1 mRNA and protein levels, whereas VGLUT2 and VIAAT/VGAT were significantly down-regulated. Treatment with the GABA_A_R blocker bicuculline (Bic) for 2 days that increased overall network activity resulted in a decrease of VGLUT1, but in an enhancement of VGLUT2 and VIAAT/VGAT expression. These activity dependent changes in expression of transporters are reflected in a modulation of their synaptic abundance (De Gois et al., 2005; Lazarevic et al., 2011). It was demonstrated that over-expression of VGLUT1 in mammals or DVGLUT in *Drosophila* led to its increased incorporation into the SV membrane resulting in increased glutamate loading and release per vesicle (Daniels et al., 2004; Wilson et al., 2005). Therefore, activity-dependent modulation of transporter expression also leads to the differences in vesicular loading and consequently to modulation of quantal size (Wilson et al., 2005). In *Drosophila*, an over-expression of DVGLUT was compensated by a decrease in number of released vesicles (Daniels et al., 2004), which suggested the existence of a homeostatic mechanism that compensates for excessive excitation due to increase in glutamate release and is reminiscent to presynaptic muting described in mammals (see “Molecular mechanisms of presynaptic muting”).

Vesicular glutamate transporter 1 and VGLUT2 being oppositely regulated by activity are expressed in distinct neuronal populations, which are only partially overlapping, and it is possible that differential regulation of both glutamate transporters plays a role in homeostatic shaping of circuit function. It was suggested that VGLUT2 is preferentially expressed at excitatory synapses contacting inhibitory neurons. Thus an activity-dependent regulation of VGLUT2 (Doyle et al., 2010) and VIAAT/VGAT, with opposite magnitude comparing to VGLUT1, might represent an intrinsic way for neurons to adjust their vesicular transmitter stores that are available for release to maintain or restore the E/I balance (De Gois et al., 2005). In agreement, a reduction of inhibitory transmission due to reduced SV filling by GABA was demonstrated upon chronic activity inactivation in cultured hippocampal neurons (Hartman et al., 2006).

Not only vesicular transporters but also enzymes involved in neurotransmitter synthesis might be regulated to contribute to presynaptic homeostatic adaptation. Lau and Murthy (2012) demonstrated recently that expression of glutamic acid decarboxylase 67 (GAD67), which is the rate-limiting enzyme in GABA synthesis, is regulated by global network activity and that this regulation affects the filling of SVs with neurotransmitter. Chronic suppression of activity with TTX resulted in decrease of GAD67 expression, reduced levels of GABA and lower mIPSC. Opposite changes in GAD67 expression and GABA levels were observed when network activity was elevated by prolonged mild depolarization of neurons or by treatment with GABA_A_R blocker picrotoxin. (Lau and Murthy, 2012)

Taken together, expression of vesicular transporters and enzymes involved in the synthesis of neurotransmitters regulates the levels of released neurotransmitters and therefore these proteins are critical determinants for the scaling of quantal size within physiological limits.

## MODIFICATION OF RELEASE APPARATUS BY HOMEOSTATIC SYNAPTIC PLASTICITY

The alterations in RRP are the hallmark of virtually all forms of homeostatic adaptation at presynapse. Despite of their repetitive description, the underlying molecular mechanisms are not fully understood. One of the first described molecular changes correlating with an enhancement of presynaptic activity upon chronic blockade of glutamate receptors was the down-regulation of the interaction between synaptophysin and synaptobrevin/vesicle-associated membrane protein 2 (VAMP2; Bacci et al., 2001). Synaptophysin was suggested to bind synaptobrevin/VAMP2 and thereby hinder it to assemble into SNARE fusion complex required for exocytosis (Edelmann et al., 1995).

Several studies reported regulation of expression levels of synaptic proteins during presynaptic homeostatic adaptation to alterations in global network activity (Thiagarajan et al., 2005; Cohen et al., 2011; Lazarevic et al., 2011; Weyhersmuller et al., 2011). This suggested that functional presynaptic alterations might be connected with molecular remodeling of the release machinery. The main candidates for regulation were proteins functioning in neurotransmitter release such as SV proteins, Cavs and components of cytomatrix at the active zone (CAZ) implicated in the regulation of presynaptic release (Gundelfinger and Fejtova, 2012; Sudhof, 2012). We tested systematically changes in the expression of these proteins using quantitative immunoblotting and immunostainings at the level of single synapses (Lazarevic et al., 2011). In our experiments, we found a significant up-regulation of synaptic vesicle proteins, which is in good agreement with increased SVP upon network inactivation reported previously (Murthy et al., 2001). On the other hand, inactivity induced by prolonged (48-h) blockage of glutamate receptors resulted in the down-regulation of cellular expression levels of presynaptic scaffolding proteins bassoon, piccolo, ELKS/CAST, Munc13, RIM, liprin-α, and synapsin. This was accompanied by a general reduction of bassoon, piccolo, ELKS/CAST, Munc13 and synapsin levels at individual synaptic sites, whereas RIM was up-regulated in a subpopulation of synapses suggesting its redistribution upon activity withdrawal (**Figure 1B**). Interestingly, the amounts of RIM correlated well with activity levels, when analyzed at individual synapses, suggesting a role of RIM in defining the presynaptic probability of SV release in normally active and silenced cultures (Lazarevic et al., 2011). RIM is a multifunctional protein regulating the RRP size likely by binding to the priming factor Munc13, what in turn leads to release of Munc13 from an autoinhibitory complex and its activation (Deng et al., 2011). The key role of RIM in regulating the RRP during homeostatic plasticity was also confirmed by studies in *Drosophila* (Muller et al., 2012) and in studies on mechanisms of synaptic muting (Jiang et al., 2010), which will be discussed later.

Using combined imaging of a genetically encoded Ca^2^^+^ reporter localized on the synaptic vesicles and a reporter of synaptic vesicles fusion, Zhao et al. (2011) showed that a decrease in the network activity in cultured hippocampal neurons causes an increase in the amount of Ca^2^^+^ entry into presynaptic boutons and an increase in Pr. Furthermore, they found a third-power relation between homeostatic changes in presynaptic Ca^2^^+^ influx and Pr, proposing that even small changes in the number and/or function of presynaptic Ca^2^^+^ channels might have large effects on synaptic strengths (Zhao et al., 2011). Accordingly, our data revealed an accumulation of the pore-forming subunit Cav2.1 of P/Q-type Cavs at the synapses of activity-deprived neurons (Lazarevic et al., 2011). Recently, a novel role for RIM proteins in the localization of Cavs to the AZ was described (Han et al., 2011; Kaeser et al., 2011) and it is well possible that the homeostatic plasticity-induced changes in Ca^2^^+^ influx are dependent on RIM-mediated recruiting of Cavs to release sites. Moreover, the inactivity also induces enrichment of the Ca^2^^+^ sensor protein synaptotagmin1 (Lazarevic et al., 2011), which was also described to interact with RIM (Coppola et al., 2001; Schoch et al., 2002). Thus, RIM might contribute to the manifestation of homeostatic adaptations not only by controlling the vesicle priming but also by recruiting multiple interaction partners involved in coupling of release sites to Ca^2^^+^ signaling.

The extensive molecular remodeling of the release machinery induced by activity withdrawal requires regulation of protein turnover at synapses, which might be in principal driven by two mechanisms: (1) alteration of protein synthesis rates at transcriptional or translational level or (2) regulation of the selective removal of synaptic proteins, mostly via the ubiquitin-proteasome system (UPS; **Figures 1B,C**).

Synaptic vesicle glycoprotein 2A, a protein associated with SV and involved in priming process (Chang and Sudhof, 2009), has been recently shown to be a target of regulation by the microRNA miR-485 (Cohen et al., 2011). The expression levels of SV2A are reduced after chronic elevation of synaptic activity. Interestingly, inhibition of miR-485 function interferes with SV2A down-regulation indicating that the control of stability of certain mRNAs by microRNAs contributes to homeostatic adaptation. Although there are no concrete targets known yet, the regulation of gene expression at the level of transcription likely plays a role in the presynaptic homeostatic plasticity as inhibition of transcription interferes with increase in mEPSC frequency upon network activity silencing (Han and Stevens, 2009).

The UPS is emerging as a powerful modulator of synaptic function, acting at both postsynaptic and presynaptic sites (Hegde, 2010). Moreover, UPS was shown to regulate the turnover of presynaptic CAZ proteins RIM (Yao et al., 2007), Munc13 (Speese et al., 2003) and liprin-alpha (van Roessel et al., 2004). Recently, bassoon and piccolo were identified to control levels of presynaptic ubiquitination, which is at least partially mediated by their interaction with E3 ubiquitin ligase SIAH-1 leading to enzyme inhibition. Consequently, loss of these two major CAZ components promotes excessive ubiquitination and degradation of many presynaptic proteins, which results in synapse degeneration (Waites et al., 2013). We tested involvement of UPS in inactivity-induced down-regulation of CAZ proteins and demonstrated that UPS-driven degradation is highly substrate specific and that it is controlled by network activity. In our study, UPS-dependent degradation of bassoon and liprin-alpha was enhanced upon activity deprivation, but interestingly, RIM and Munc13 were unchanged (Lazarevic et al., 2011). These results suggest that alternative cellular mechanisms control the redistribution of RIM in response to activity withdrawal. However, as it will be discussed later RIM seems to be a target of UPS-mediated degradation during presynaptic muting (Jiang et al., 2010).

Taken together, intense molecular reorganization of release sites underlies the functional alteration of release during homeostatic presynaptic adaptation. Although, several effector proteins regulated during this process have been identified, the underlying signaling pathways and exact nature of their modification needs to be addressed by future studies.

## MOLECULAR MECHANISMS OF PRESYNAPTIC MUTING

Adaptive presynaptic silencing or presynaptic muting is a form of presynaptic homeostatic adaptation preventing the runaway excitation or excitotoxicity during physiological and pathophysiological depolarization such as seizures or hypoxic insults. Presynaptic muting can be induced by rearing cultured dissociated hippocampal neurons or cerebellar granule neurons at elevated K^+^ concentrations. It is manifested by selective functional inactivation of excitatory presynapses, which appear normal if assessed morphologically (Moulder et al., 2004, 2006). Presynaptic muting is due to a decrease in size of the RRP at individual AZs and does not require glutamate receptor activation or rises in intracellular Ca^2^^+^ from neither extracellular nor intracellular stores (Moulder et al., 2006; Crawford et al., 2011). The reduction of RRP is likely due to a block in the priming process as treatment with alpha-latrotoxin induces neurotransmitter release in muted synapses (Moulder et al., 2006). In parallel to the effect on RRP, prolonged depolarization induces depressed Pr, which is common to both glutamate and GABA transmission and likely due to reduced Ca^2^^+^ influx into presynaptic terminals (Moulder et al., 2003, 2004). The effects on Pr and RRP seem to be independent as their manifestation is separated temporally (with changes in the RRP seen already 4 h, but Pr only 16 h after depolarization) and segregated between glutamatergic and GABAergic synapses, whereas only the glutamatergic synapses express the muting (Moulder et al., 2004). Under physiological conditions a certain fraction of synapses remains unresponsive (silent) to action potentials and this fraction can be modulated in response to changes in physiological activity. This suggests that presynaptic muting also takes place under physiological conditions and might represent a mechanism, by which neuronal networks tune the excitatory drive depending on the levels of network activity (Moulder et al., 2006). Blockade of action potential propagation could reverse depolarization-induced muting, whereas block of glutamate receptors could not, what suggests a presynapse autonomous mechanism (Moulder et al., 2006).

Only a subset of synapses in depolarized cultures exhibit presynaptic muting and it is unknown, what are the molecular determinants of this variability. Presynaptic muting is reminiscent of the synaptic phenotype of mouse mutants for the CAZ proteins Munc-13 (Augustin et al., 1999; Rosenmund et al., 2002) and bassoon (Altrock et al., 2003), which display a reduction in numbers of functional synapses and deficits in SV priming. This suggests an involvement of CAZ proteins in the process of presynaptic muting. This assumption is supported by the finding that levels of CAZ proteins RIM and Munc-13 are decreased in muted synapses as compared to active ones, both under basal conditions and after depolarization induced synaptic muting (Jiang et al., 2010). The reduction of RIM expression levels at synapse are due to its degradation by the UPS; inhibition of UPS by the proteasomal blocker MG-132 fully inhibited the depolarization-induced decrease in RIM and also prevented presynaptic muting (**Figure 1C**). In line with this notion, over-expression of RIM1 completely prevented depolarization-induced synaptic muting as well as decrease of Munc-13 levels (Jiang et al., 2010) supporting previous assumptions on a function of RIM in maintaining the Munc13 synaptic levels (Schoch et al., 2002). Taken together, these experiments demonstrate that the modulation of RIM1 expression levels at synapse play a key role in the induction of presynaptic muting and also suggest an important role of the UPS in this process.

Cyclic-AMP (cAMP) signaling was also proposed to play a role in presynaptic muting as a treatment with forskolin, an activator of adenylate cyclases (AC) inducing an increase of cAMP levels, prevented induction of synaptic muting upon depolarization and reduced the fraction of muted synapses under basal conditions (Moulder et al., 2008). This pathway seems to interfere with the UPS in a more complex manner; forskolin treatment did not affect proteasomal activity but did affect synaptic levels of RIM1 and Munc13 (Jiang et al., 2010). To identify the molecular components of the cAMP signaling pathway involved in presynaptic muting, a requirement of the two main Ca^2^^+^-sensitive ACs (types 1 and 8) was tested. The activity these two ACs is not needed to induce muting, but recovery from depolarization induced muting was strongly affected in neurons derived from knockout mice for AC8. The treatment with a broadly acting inhibitor of cAMP-dependent kinases or with specific PKA inhibitor, however, completely prevented recovery from presynaptic muting suggesting a contribution of multiple forskolin-sensitive ACs to control this process (**Figure 1C**; Moulder et al., 2008). In a follow-up study on this topic, presynaptic muting was completely prevented by pharmacological blockade of inhibitory G (G_i/o_) proteins. The exact target of this treatment was not identified, but it was shown that activation of adenosin A1 and GABA_B _receptors induced presynaptic muting, which was dependent on normal activity of the UPS. However, blockade of these receptors did not interfere with the induction of presynaptic muting by depolarization (Crawford et al., 2011).

Recovery from presynaptic muting can be induced by shifting neurons to media with physiological K^+^ concentration, which leads to the establishment of normal membrane potential. Such “unmuting” is detectable after 3 h recovery, requires PKA activity (Moulder et al., 2008) and is blocked in the presence of transcription and translation inhibitors suggesting a role of protein synthesis in this process (Crawford et al., 2012b). Potential protein candidates synthesized during unmuting are RIM and Munc13. Their levels are reduced in muted synapses, recover during unmuting, but remain reduced if translation and transcription are blocked (Crawford et al., 2012b). It is not yet fully understood how PKA activation translates into regulation of protein synthesis. PKA regulates activity of CREB during unmuting (Crawford et al., 2012b), but it is likely that other transcription factors are also involved. Fast synaptic unmuting can also be induced by treatment with phorbol esters and is measurable within several minutes (Moulder et al., 2008; Chang et al., 2010). It is not dependent on proteins synthesis and does not require PKC activity. Likely it depends on direct interaction of phorbol esters with Munc13-1, one of the main priming/docking factors (Betz et al., 1998; Chang et al., 2010). Thus studies on presynaptic muting and unmuting propose a role of cAMP signaling in these processes, which results in tuning of turnover of key regulators of Pr, i.e., RIM and Munc13, and involves modulation of protein synthesis as well as protein degradation by UPS.

Recently, it was demonstrated that astrocyte deprivation prevented presynaptic muting. The role of thrombospondins, glycoproteins secreted by astrocytes, was proposed. Gabapentin, a high affinity antagonist of thrombospondins binding to its receptor α2δ, mimicked effect of astrocyte deprivation (Crawford et al., 2012a). α2δ functions also as an auxiliary subunit of Cavs and regulates their synaptic localization and function (Dolphin, 2012). Astrocyte deprivation leads to abnormal activity of PKA and increased phosphorylation of its target proteins such as synapsin, cAMP response element-binding protein (CREB). However, the link between α2δ-1 and PKA signaling remains unclear (Crawford et al., 2012a). This study put forward a novel mechanism, by which neuron - glia interaction might control synaptic homeostasis.

## PRESYNAPTIC HOMEOSTATIC PLASTICITY AND BRAIN DISEASE

Numerous brain dysfunctions are connected with disturbed physiological synaptic functions leading to changes in the activity of brain circuits, which in turn induces maladaptive plasticity. The mechanisms of induction and the consequences of such maladaptive plasticity closely relates to homeostatic plasticity.

Dickman and Davis (2009) demonstrated a critical role of *dysbindin*, a gene linked to schizophrenia in humans, in homeostatic presynaptic adaptations in *Drosophila *NMJ. In vertebrates, dysbindin is associated with SVs (Talbot et al., 2006) and is involved in the regulation of glutamate release (Numakawa et al., 2004). In schizophrenia patients, dysregulation of glutamatergic transmission and reduced expression of dysbindin were reported (Mechri et al., 2001; Talbot et al., 2004, 2006; Weickert et al., 2004, 2008; Tang et al., 2009), suggesting that dysfunction of dysbindin may result in failure of homeostatic plasticity mechanisms tuning glutamatergic transmission.

Presynaptic muting was proposed to play a key role in protecting neurons from damage due to glutamate excitotoxicity occurring during epileptic seizures, hypoxia or ischemia (Hogins et al., 2011). The preconditioning by mild depolarization, which induces synaptic muting by UPS-dependent mechanisms (Moulder et al., 2004; Jiang et al., 2010), protected neurons from damage induced by hypoxia or oxygen/glucose deprivation. Blockade of UPS during preconditioning completely abolished the preconditioning induced protection. Hypoxia itself induced muting in a proteasome-dependent manner; UPS inhibition exacerbated neuronal loss upon mild hypoxia and prevented hypoxia-induced muting. These data suggest that overexcitation-induced presynaptic muting provides endogenous neuroprotection mechanisms to limit the damage from insults involving excess synaptic glutamate release (Hogins et al., 2011).

Several presynaptic proteins involved in the homeostatic regulation of neurotransmitter release were linked to epilepsy, where excessive synaptic activity occurs leading to glutamate-induced excitotoxicity. Mice mutant for the SV protein SV2A exhibit spontaneous epileptic seizures (Crowder et al., 1999; Janz et al., 1999) similarly as do mice mutant for synapsin (Rosahl et al., 1995) or the AZ protein bassoon (Altrock et al., 2003) suggesting important roles of these proteins in the control of baseline neuronal activity at the organismal level. Interestingly, a recent study has shown that mice mutant for protein RIM1aplha show a dramatically increased frequency of spontaneous recurrent seizures upon experimental induction of epilepsy, despite of the fact that these mice do not show any changes in the basal activity patterns assessed by EEG (Pitsch et al., 2012). This phenotype is consistent with the proposed key role of RIM regulation in the induction of presynaptic muting (Jiang et al., 2010) and with the idea that presynaptic homeostatic plasticity mechanisms such as presynaptic muting are recruited for dampening synchronous activity-induced epileptogenesis.

Homeostatic synaptic plasticity, which constantly acts to stabilize neuronal networks whenever Hebbian plasticity or pathological synapse dysfunction alter the synapse weight, turned out to be a key mechanism ensuring the long-term functioning of brain circuits. Accordingly, cellular mechanisms controlling homeostatic synaptic plasticity, as well as underlying molecules and signaling pathways represent emerging targets for drug development and new therapeutic strategies for various neurological and psychiatric disease conditions.

## Conflict of Interest Statement

The authors declare that the research was conducted in the absence of any commercial or financial relationships that could be construed as a potential conflict of interest.

## AUTHOR CONTRIBUTIONS

Vesna Lazarevic, Santosh Pothula, Maria Andres-Alonso and Anna Fejtova wrote the paper, Vesna Lazarevic prepared the figure. Vesna Lazarevic, Santosh Pothula and Maria Andres-Alonso contributed equally to this work

## Abbreviations

AC: adenylate cyclase
AMPA: alpha-amino-3-hydroxy-5-methyl-isoxasole-4-propionic acid
AZ: active zone
Bic: bicuculline
cAMP: cyclic adenosine monophosphate
CASK: calcium/calmodulin-dependent serine protein kinase
Cav: voltage-dependent calcium channel
CAZ: cytomatrix at the active zone
CDK5: cyclin-dependent kinase 5
CNB: calcineurin B
CREB: cAMP response element-binding protein
DVGLUT: *Drosophila* vesicular glutamate transporter
E/I: excitation/inhibition
EEG: electroencephalography
GABA: gamma-aminobutyric-acid
GAD67: glutamic acid decarboxylase 67
mEPSC: miniature excitatory postsynaptic current
mIPSC: miniature inhibitory postsynaptic current
Munc-13: mammalian homologues of the *C. elegans* unc-13 gene
NBQX: 1,2,3,4-tetrahydro-6-nitro-2, 3-dioxo[f]quinoxaline-7-sulfonamide disodium
NMJ: neuromuscular junction
PhTx: philantotoxin-433
PKA: protein kinase A
PKC: protein kinase C
Pr: release probability
RIM: rab3-interactning molecule
RP: recycling pool
RRP: readily releasable pool
RtP: resting pool
SV: synaptic vesicle
SVP: synaptic vesicle pools
SV2A: synaptic vesicle glycoprotein 2A
TRP: total recycling pool
TTX: tetrodotoxin
UPS: ubiquitin-proteasome system
VAMP-2: vesicle-associated membrane protein 2
VGAT: vesicular GABA transporter
VGLUT: vesicular glutamate transporter
VIAAT: vesicular inhibitory amino acid transporter
